# Free Fatty Acids in CSF and Neurological Clinical Scores: Prognostic Value for Stroke Severity in ICU

**DOI:** 10.1155/2020/5808129

**Published:** 2020-07-17

**Authors:** Sayed Gaber, Sherine Ibrahim ElGazzar, Mahmoud Qenawi, Nora Ismail Mohamed Abbas

**Affiliations:** Critical Care Medicine, Critical Care Department, Faculty of Medicine, Cairo University, Giza, Egypt

## Abstract

**Introduction:**

Brain ischemia initiated significant increase in FFAs in animal studies. Accumulation of FFA can lead to liberation of inflammatory byproducts that contribute to neuronal death. Increased risk of systemic thromboembolism was seen in animal models after FFA infusion possibly through activation of factor XII by stearic acids. The clinical studies that examined the relation between stroke in humans and CSF biomarkers are infrequent. *Aim of Work*. We tried to evaluate the potential role of FFAs in CSF in the diagnosis and the prognosis of ICU patients with AIS while comparing the results to traditional neurological scoring systems. *Patients and Methods*. Our study included 80 patients who were admitted to ICU with acute ischemic stroke (AIS) within 24 hours of the onset of cerebral infarction. CSF samples were obtained at admission. The FFA levels were measured using the sensitive enzyme-based colorimetric method. The NIHSS, GCS, and mRS were evaluated at admission and at 30 days. Univariate and multivariate analysis were used to evaluate the stroke outcome according to FFA levels in CSF.

**Results:**

Worsening of the GCS (<7) at 30 days showed a significant correlation with FFA in CSF. The ROC curve showed a cutoff value of 0.27 nmol/*µ*l, sensitivity of 62.9%, and specificity of 72.2%. There was a significant correlation between FFA in CSF and the mRS >2 at 30 days. The ROC curve showed a cutoff value of 0.27 nmol/*µ*l, specificity of 69.2%, and sensitivity of 59.7%. There was a significant correlation between FFA in CSF and the NIHSS ≥ 16 at 30 days. The ROC curve showed a cutoff value of 0.27 nmol/*µ*l, specificity of 72.2%, and sensitivity of 62.9%. Our study subdivided patients according to infarction volume and compared the 2 subgroups with FFA in CSF. We found a significant difference between 2 subgroups. FFA levels showed a positive correlation with infarction volume ≥145 ml. The ROC curve showed a cutoff value of 0.25 nmol/*µ*l, sensitivity of 76.9%, and specificity of 71.4%. Our study showed that FFA in CSF was a significant predictor of all-cause mortality (0.37 + 0.26, *P* value 0.007). The ROC curve showed a cutoff value of 0.27, specificity of 72.2%, and sensitivity of 62.9%. There was a positive correlation between FFA in CSF and neurological causes of mortality (0.48 + 0.38, *P* value 0.037). The ROC curve showed a cutoff value of 0.37 nmol/*µ*l, specificity of 76.1%, and sensitivity of 61.5%.

**Conclusion:**

FFA in CSF may serve as an independent prognostic biomarker for assessing the prognosis of acute ischemic stroke and the clinical outcome. It might be a useful biomarker for early detection of high-risk patients for poor outcome and hence more aggressive treatment.

## 1. Introduction

Acute ischemic stroke (AIS) is diagnosed by clinical examination after exclusion of mass lesion or intracranial hemorrhage by CT or MRI. Treatment of intracranial complications, reperfusion strategies, monitoring of biological and vital parameters, and optimization of homeostasis are some indications for patients with AIS to be admitted to intensive care unit (ICU) [1].

Brain ischemia initiated significant rises in FFAs in animal studies [2].

Accumulation of FFA can lead to uncoupling of oxidative phosphorylation and liberation of inflammatory by-products including reactive oxygen species that contribute to neuronal death [3].

Increased risk of systemic thromboembolism was seen in animal models after FFA infusion. A possible mechanism is activation of factor XII by stearic acids [4].

Brain biomarkers might help in the diagnosis and management of AIS [5].

With the presence of blood-brain barrier, the ideal biomarker for brain injury should be brain specific, appears very early, and the peak level reveals the severity and suggests the neurological outcome [6].

The clinical studies that examined the relation between the stroke in humans and CSF biomarkers are infrequent.

### 1.1. Aim of Work

We tried to evaluate the potential role of FFAs in CSF in the diagnosis and the prognosis of ICU patients with AIS while comparing the results to traditional neurological scoring systems.

### 1.2. Patients and Methods

Our study included 80 patients with acute ischemic stroke (AIS) (group 1) who were admitted to ICU within 24 hours of the onset of cerebral infarction and whose lesion was confined according to neurological examination and computed tomography (CT) or magnetic resonance imaging (MRI) in comparison to 28 age- and sex-matched healthy subjects served as the control (group 2).

The study was conducted in critical care unit at Shebin El Kom Teaching Hospital in the period between October 2016 and May 2017.

#### 1.2.1. Exclusion Criteria

- Age less than 18 years
- Strokes that are due to nonvascular cause (e.g., tumor, trauma, infection, hypoglycemia, and peripheral lesion) or was an extension of a previous stroke
- Transient ischemic attack (TIA), intracerebral hemorrhage (ICH), and subarachnoid hemorrhage (SAH)
- Patients treated with a recombinant tissue plasminogen activator

Included patients were subjected to the following:

(i) History taking and clinical examination including general and neurological examinations.
(ii) Routine laboratory investigations.
(iii) Brain imaging, either CT or MRI brain. The infarct size was estimated according to the rules used by Sims et al. [7] (size = 0.5 × *a* × *b* × *c*) where *a* is the maximal longitudinal diameter, *b* is the maximal transverse diameter perpendicular to a, and *c* is the number of 10 mm slices containing infarction, and lesions were classified according to their sizes as follows:
  - Small to medium, when size is less than 145 ml
  - Large, when size is equal to or more than 145 ml [8, 9]
(iv) The Glasgow Coma Scale (GCS) at admission and 30 days [10].
(v) Stroke severity being assessed by the National Institutes of Health Stroke Scale (NIHSS) score at admission and 30 days [11].

Patients were classified according to stroke severity; more than 20 is considered severe stroke [12].

#### 1.2.2. Outcome Measurements

Patients were prospectively followed up for the following outcomes:

- In-hospital or 30-day mortality
- The modified Rankin Scale was used to assess functional disability and was evaluated at admission and at one month from stroke onset [13]

The patients were divided according to the modified Rankin Scale score into the following categories:

- Good outcome (score 0 to 2)
- Dependent or dead (score 3 to 6) [14]

#### 1.2.3. Free Fatty Acids Level Measurement in CSF

CSF samples were obtained within 24 hours from admission under complete aseptic conditions. The FFA levels were measured using the sensitive enzyme-based colorimetric method.

### 1.3. Statistical Methods

Data were coded and entered using the statistical package SPSS (Statistical Package for the Social Sciences) version 24. Data were summarized using mean, standard deviation, median, minimum and maximum in quantitative data, and using frequency (count) and relative frequency (percentage) for categorical data. Comparisons between quantitative variables were done using the nonparametric Mann–Whitney test. For comparison of serial measurements within each patient the nonparametric Friedman test and Wilcoxon signed rank test were used [15]. For comparing categorical data, the chi square (*χ*^2^) test was performed. The exact test was used instead when the expected frequency is less than 5 [16]. Correlations between quantitative variables were done using Spearman correlation coefficient [17]. The ROC curve was constructed with area under curve analysis performed to detect the best cutoff value of CSF FFA for detection of mortality, severity, and functional outcome. *P* values less than 0.05 were considered as statistically significant.

## 2. Results

There was a highly significant difference between the patients group and the control group concerning FFA concentration in CSF (Table 1).

### 2.1. Free Fatty Acids in CSF and the Glasgow Coma Scale

The GCS at 30 days shows a significant correlation with FFA in CSF (Table 2).

### 2.2. Free Fatty Acids in CSF and the Modified Rankin Scale

There was a significant correlation between FFA in CSF and the mRS >2 at 30 days (Table 3).

### 2.3. Free Fatty Acids in CSF and the National Institutes of Health Stroke Scale

There was a significant correlation between FFA in CSF and the NIHSS ≥ 16 at 30 days (Table 4).

### 2.4. Correlation between Free Fatty Acids in CSF and Infarction Size

Our study subdivided (group 1) patients according to infarction volume and compared the 2 subgroups with FFA in CSF. We found significant difference between 2 subgroups (Table 5).

### 2.5. Free Fatty Acids in CSF as a Predictor of All-Cause Mortality

Our study showed that FFA in CSF was a significant predictor of all-cause mortality in (group 1) patients (0.37 + 0.26, *P* value 0.007) (Table 6).

### 2.6. Free Fatty Acids in CSF and Neurological Causes of Mortality

There were significant positive correlations between FFA in CSF and neurological causes of mortality (*P* value 0.037) (Table 8).

## 3. Discussion

Our study results revealed that the mean value of FFA levels in CSF at admission was 0.34 + 0.24 nmol/*µ*l in the patients group and 0.22 + 0.04 nmol/*µ*l in the control group, which reflects significant elevation of FFA levels in CSF in patients with AIS compared to healthy controls (*P* < 0.001).

These findings were consistent with Sun et al. [18] who stated that the median FFA levels in CSF were significantly higher in patients with stroke compared to controls (*P* < 0.0001).

Our study reported a significant linear correlation between FFA in CSF and the mRS >2 at 30 days (*P* value 0.037). These results suggested that FFA in CSF might be a helpful predictor of outcome in AIS.

Similar results were seen with Wang et al. [19] when they studied 217 patients with AIS. They reported that high levels of FFA in CSF were associated with poor prognosis (defined as a mRS score of 3–6) and stroke recurrence. They suggested that FFA in CSF can have a predictive value for the prognosis and outcome in AIS.

Also, Wei et al. [20] stated that the FFA level in CSF could be an independent predictive indicator for functional outcome up to 90 days after AIS.

Likewise, Pilitsis et al. [2] reported higher concentrations of polyunsaturated fatty acids (PUFAs) in CSF collected within 48 hours of AIS and were linked to poor outcome at hospital discharge (*P* < 0.01).

In our study, we found a statistically significant linear correlation between FFA in CSF and the NIHSS ≥ 16 at 30 days after the onset of stroke (*P* value 0.049).

Moreover, we found a statistically significant linear correlation between FFA in CSF and the GCS < 7 at 30 days from stroke onset (*P* value 0.048). These results suggested that FFA in CSF could be a respectable prognostic predictor of severity in AIS.

This was in agreement with Wei et al. [20] who studied 238 patients with AIS between 2012 and 2014; then, he found that elevated FFA levels in CSF were correlated with increasing severity of stroke after accounting for the NIHSS score.

In the same way, Sun et al. [18] assessed FFA levels in CSF at 4 time points, and the severity of stroke was estimated with the NIHSS score from December 2011 to October 2014. They concluded that FFA levels increased as the severity of stroke increased.

Also, Niu et al. [21] studied 296 patients from December 2013 to May 2015 with AIS. NIHSS was measured at the time of admission in addition to FFA levels in CSF. They found a significant correlation between FFA levels in CSF and the NIHSS score as well as stroke recurrence (*P* < 0.0001).

In our study, we found a statistically significant correlation between FFA levels and all-cause mortality. In patients who died, FFA levels were 1.5 folds greater compared to patients who survived (mean 0.37 + 0.26 nmol/*µ*l vs. 0.24 + 0.6 nmol/*µ*l) (*P*=0.007), with a cutoff value of 0.27, specificity of 72.2%, and sensitivity of 62.9%.

Also with multivariate analysis, there was an increased risk of death associated with FFA levels ≥0.27 nmol/*µ*l (OR 7.27, 95 % CI 1.48–35.68; P 0.015) after adjusting for other confounders such as age, previous stroke, HTN, and the NIHSS, with a sensitivity of 93.44% and specificity of 61.11%. These results indicated that FFA in CSF might be a useful predictor of mortality.

In association with our results, Wei et al. [20] and Duan et al. [22] found that nonsurvivors had significantly higher FFA levels in CSF than survivors with increased risk of death.

Our results additionally showed a significant correlation between FFA levels in CSF and neurological causes of mortality with a statistically significant difference between survivors and nonsurvivors due to neurological insult as a cause of death.

This supports the suggestion that the local increase of FFA in CSF is related to neurological insult, and hence its impact on mortality.

Large volume infarction conveys increased risk of worsening of neurologic status, incapacity, stroke severity, and even death. Our results showed a significant correlation between FFA and infarction volume ≥145 ml (*P* value 0.001).

This was in agreement with [18, 19, 21]. They found a positive correlation between of FFA levels in CSF and the infarct volume.

On the other hand, our results showed no correlation between FFA levels in CSF and patients with AF (*P* value 0.277).

This was in agreement with Khawaja et al. [23], as they found no consistent increase between FFA levels and cardioembolic (CE) stroke.

Another point of view, our data were not in agreement with Wei et al. [20] and Wang et al. [19] who found a statistical correlation between cardioembolic (CE) stroke and FFA levels in CSF.

Broader study population might be required to determine the correlation between FFA in CSF and the pathogenesis of CE stroke as our study included only 20 patients with AF.

We also reported higher FFA levels in patients with AIS who had previous stroke (*P* value 0.048). This supports the prognostic role of FFA in CSF and the evidence that persistent elevation after stroke episode brings risk of stroke repetition.

The baseline FFA levels could be influenced by a number of factors, such as infections and myocardial ischemia through catecholamine-induced responses, in addition to hormones (e.g., insulin) and blood lipids. Unfortunately, in our study, we could not exclude definitely these conditions.

Nakano et al. [24] detected an escalation of FFA levels in the gerbil brain after 5 minutes of ischemia, reached a peak within 3 days, and then decreased again.

In our study, we assessed FFA in CSF only at admission, so the follow-up data regarding the peak level and duration of elevation are missing.

We tested FFA only in CSF without plasma samples, and this was performed after the stroke event, which might not accurately represent prestroke status and the relation between CSF and plasma levels.

FFAs level in CSF can provide important therapeutic applications:

- It has been shown that normalization of plasma FFA levels with acipimox, a nicotinic analog, normalized insulin resistance in obese, nondiabetic subjects and improved insulin resistance in obese patients with T2DM. Nicotinic acid or long-acting nicotinic acid analogs effectively lower plasma FFA levels. Unfortunately, their use is associated with a rebound of plasma FFA to very high levels, which makes this class of drugs unsuitable for the long-term control of plasma FFA.
- Thiazolidinediones (TZD) lower plasma FFA levels for long-term and without rebound. They do this primarily by stimulating fat oxidation through a coordinated induction of genes in the adipose tissue related to FFA uptake, binding, *β*-oxidation, and oxidative phosphorylation [25]. However, TZD-mediated lowering of plasma FFA levels is modest, ranging from <10% to 20%. Moreover, several side effects limit its use [26].
- Fibrates, another class of lipid-lowering drugs also lower plasma FFA levels modestly and without rebound primarily by stimulating fat oxidation in the liver [27]. As both classes of drugs work in different organs (TZDs in fat and fibrates in the liver) and through different mechanisms (TZDs through activation of PPAR-*γ* and fibrates through activation of PPAR-*α*), their use in combination produces greater decreases in plasma FFA levels as well as greater improvements in insulin sensitivity than the use of either drug alone [28].
- Lowering of plasma FFA, in addition to improving insulin sensitivity, may also prevent activation of the proinflammatory and proatherogenic NF*κ*B pathway and thus may reduce the incidence of atherosclerotic vascular problems.
- The use of such medications or others needs further studies to weigh their efficacy especially in patients with AIS and their role either as prophylactic or therapeutic.

Contraindications for lumbar puncture include candidates for thrombolysis and severe brain injury with increased intracranial tension (ICP). Those patients were excluded from our study. Hypothetically, if CSF samples were taken from such patients, their results could affect our results in a way or another.

Our study population was relatively small in size (only 80 patients), while larger sample size population might be needed for better analysis of statistics and data validation in a long-term prospective cohort study.

Also, we measured all-cause mortality; however, categorization of death is sometimes unreliable in clinical practice.

Despite the special training needed to apply the NIHSS, still there is a remarkable interobserver variability [29]. Also, it is less reliable in patients with posterior circulation syndromes compared to anterior circulation syndromes, and left hemispheric strokes show greater NIHSS scores than right [30].

Hence, the addition of FFA in CSF to the traditional clinical scores might have better prognostic accuracy.

## 4. Conclusion

FFA in CSF may serve as an independent prognostic biomarker for assessing the prognosis of acute ischemic stroke and its clinical outcome. It might be a useful valid surrogate biomarker for early detection of high-risk patients for poor outcome and hence more aggressive treatment.

## Figures and Tables

**Figure 1:**
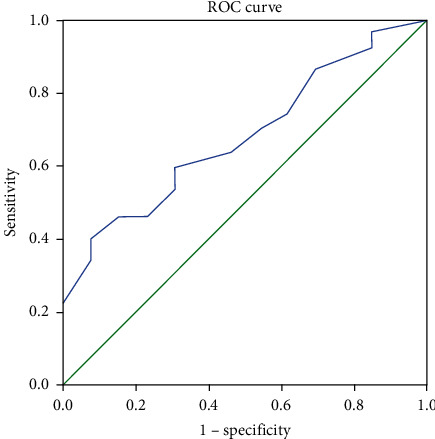
ROC curve for correlation between FFA in CSF and mortality. Diagonal segments are produced by ties.

**Figure 2:**
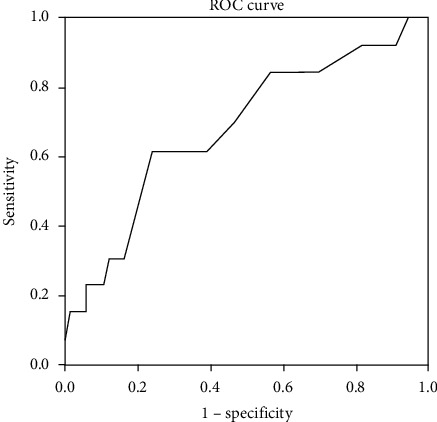
FFA in CSF and neurological causes of mortality. Diagonal segments are produced by ties.

**Table 1:** Case control comparison of FFA in CSF.

	Patients		Control		*P* value
	Mean ± SD	Range	Mean ± SD	Range	
FFA in CSF (nmol/*µ*l)	0.34 ± 0.24	0.10–1.40	0.22 ± 0.04	0.16–0.26	<0.001

Correlation between free fatty acids in CSF and clinical scores.

**Table 2:** Correlation between free fatty acids in CSF and the Glasgow Coma Scale.

		FFA in CSF			*P* value
		Mean ± SD	Median	Range	
GCS 30 days	<7	0.38 ± 0.28	0.30	0.14–1.40	0.048
	≥7	0.26 ± .10	0.26	0.10–0.46	

The ROC curve for correlation between FFA in CSF and worsening of the GCS (<7) showed a cutoff value of 0.27 nmol/*µ*l, sensitivity of 62.9%, and specificity of 72.2%.

**Table 3:** Correlation between free fatty acids in CSF and the modified Rankin Scale.

		FFA in CSF			*P* value
		Mean ± SD	Median	Range	
mRS 30 days	≤2	0.36 ± 0.25	0.30	0.10–1.40	0.037
	>2	0.24 ± 0.08	0.24	0.10–0.38	

The ROC curve for correlation between FFA in CSF and worsening of the mRS (>2) showed a cutoff value of 0.27 nmol/*µ*l, specificity of 69.2%, and sensitivity of 59.7%.

**Table 4:** Correlation between free fatty acids in CSF and the National Institutes of Health Stroke Scale.

		FFA in CSF			*P* value
		Mean ± SD	Median	Range	
NIHSS 30	≥16	0.37 ± 0.27	0.30	0.10–1.40	0.049
	<16	0.27 ± 0.10	0.26	0.10–0.46	

The ROC curve for correlation between FFA in CSF and deterioration of the NIHSS (≥16) showed a cutoff value of 0.27 nmol/*µ*l, specificity of 72.2%, and sensitivity of 62.9%.

**Table 5:** Correlation between free fatty acids in CSF and infarction volume.

	Infarction volume (ml)				*P* value
	<145		≥145		
FFA in CSF	Mean ± SD	Range	Mean ± SD	Range	0.001
	0.30 ± 0.24	0.14–1.20	0.31 ± 0.17	0.10–1.40	

CSF FFA showed positive correlation with infarction volume ≥ 145 ml. The ROC curve showed a cutoff value of 0.25 nmol/*µ*l, sensitivity of 76.9%, and specificity of 71.4%.

**Table 6:** The relation between free fatty acids in CSF and mortality.

		FFA in CSF			*P* value
		Mean ± SD	Median	Range	
Mortality	Died	0.37 ± 0.26	0.30	0.10–1.40	0.007
	Discharge	0.24 ± 0.08	0.23	0.10–0.38	

The ROC curve for correlation between FFA in CSF and mortality showed a cutoff value of 0.27, specificity of 72.2%, and sensitivity of 62.9% (Figure 1 and Table 7).

**Table 7:** AUC, *P* value, cutoff value, sensitivity, and specificity in the correlation between FFA in CSF and mortality.

AUC	*P* value	95% Confidence interval		Cutoff value	Sensitivity (%)	Specificity (%)
		Lower bound	Upper bound			
0.710	0.007	0.588	0.832	0.27	62.9	72.2

**Table 8:** Correlation between free fatty acids in CSF and neurological causes of mortality.

	Neurological causes of mortality				*P* value
	Yes (10)		No (52)		
	Mean ± SD	Range	Mean ± SD	Range	
FFA in CSF	0.48 ± 0.38	0.16–1.40	0.31 ± 0.19	0.10–1.20	0.037

The ROC curve for correlation between FFA in CSF and neurological causes of mortality showed a cutoff value of 0.37 nmol/*µ*l, specificity of 76.1%, and sensitivity of 61.5%. *P* value = 0.038 (Figure 2 and Table 9).

**Table 9:** AUC, *P* value, cutoff value, sensitivity, and specificity in the correlation between FFA in CSF and neurological causes of mortality.

Area under curve	*P* value	95% Confidence interval		Cutoff value	Sensitivity (%)	Specificity (%)
		Lower bound	Upper bound			
0.683	0.038	0.518	0.848	0.37	61.5	76.1

## Data Availability

The table data used to support the findings of this study are available from the corresponding author upon request.
